# In-Hospital Graft Occlusion in Post-Coronary Artery Bypass Grafting Patients in the Early Postoperative Period: A Systematic Review and Meta-Analysis

**DOI:** 10.3390/jcm13185514

**Published:** 2024-09-18

**Authors:** Islam Salikhanov, Luca Koechlin, Brigitta Gahl, Michael J. Zellweger, Philip Haaf, Christian Müller, Denis Berdajs

**Affiliations:** 1 Department of Cardiac, Surgery, University Hospital Basel, 4031 Basel, Switzerland; luca.koechlin@usb.ch (L.K.); denis.berdajs@usb.ch (D.B.); 2 Department of Cardiology, University Hospital Basel, 4031 Basel, Switzerland

**Keywords:** early graft occlusion, computed tomography, coronary artery bypass grafting, graft patency, postoperative imaging

## Abstract

**Objectives**: The objectives of this paper are to evaluate the incidence of early graft occlusion during hospital stays following coronary bypass surgery (CABG) and to assess the factors influencing the odds of in-hospital early graft occlusion. **Methods**: Reports evaluating the early in-hospital occlusion of coronary bypass grafts were identified through PubMed, Embase, and Cochrane databases. The primary endpoint was to determine the incidence of early graft occlusion following CABG before discharge and to identify and quantify the impact of demographic, clinical, and procedural risk factors on the occurrence of early graft occlusion. The meta-analysis was conducted using a random-effects inverse-variance model with the DerSimonian–Laird estimator, assessing incidence rates, risk factors, and study heterogeneity, with statistical analysis performed using Stata. **Results**: A total of 22 studies with 35,798 patients were included in the analysis. The overall incidence of in-hospital early graft occlusion was 5% (95% CI: 3% to 7%). In studies using symptom-driven patency assessment, the incidence of occlusion was 2%, whereas in those employing systematic graft patency assessment, it was 6%. Only the presence of a vein graft OR 2.13 (95% CI: 1.19–3.82) was significantly associated with in-hospital graft occlusion. **Conclusions**: The incidence of in-hospital early graft occlusion seems substantially underestimated if imaging is restricted only to symptomatic patients. Moreover, female gender, increased PI, and the presence of a composite graft could also be potential risk factors for this complication.

## 1. Introduction

Coronary artery bypass grafting (CABG) is a frequent procedure in treating severe coronary artery disease and is the most common cardiosurgical intervention [1,2]. Despite its routine nature, risks associated with CABG including death and periprocedural myocardial infarction may be considerable, particularly for patients with advanced cardiovascular profiles [3]. One of the most concerning but incompletely understood complications after CABG is early graft occlusion in the early postoperative period. Previous reports on the incidence of early graft occlusion have been conflicting and have provided highly variable estimates ranging from 1% to 23% [2,4,5]. In-hospital coronary graft occlusion significantly worsens both short-term and long-term clinical outcomes, manifesting as acute myocardial infarction or as reduced myocardial perfusion contributing to coronary artery disease progression, with potential for repeat revascularization and reduced survival over the mid and long term [1,2]. This underscores the importance of early assessments of coronary bypass patency and quality [6].

The scarce literature available on in-hospital bypass occlusion suggested that patient-related elements such as sex, surgical approach, and strategy may play a role [4,7]. In this matter, the quality of the bypass graft, the state of the target arteries, and the quality of the anastomoses were suggested as possible predictors for early bypass occlusion [1,2]. This systematic review and meta-analysis is the first to focus specifically on early graft occlusion during the immediate postoperative period before patient discharge. Unlike previous studies that address later occlusions, this work aims to fill the gap in understanding the critical early phase, providing insights that could improve patient outcomes during this vulnerable time.

To bridge the identified gap in the literature, the primary objective of this systematic review and meta-analysis was to consolidate all available evidence on in-hospital early coronary bypass occlusion in the early postoperative period following isolated CABG and its risk factors, aiming to enhance our understanding of this significant yet incompletely explored phenomenon.

## 2. Materials and Methods

This study is registered with PROSPERO, number CRD42024512805. The PubMed, Embase, and Cochrane databases were searched from 1 January 2004, to 1 January 2024, for full-length, English-language studies that reported on patients with early graft occlusion after CABG. We conducted a comprehensive literature search employing terms related to CABG and early graft occlusion, combining MeSH terms and keywords such as “coronary artery bypass”, “CABG”, “early graft occlusion”, “early bypass failure”, “graft thrombosis”, and their synonyms to identify studies focusing on early graft occlusion occurring before hospital discharge. The search strategy was reviewed by a dedicated information specialist. The present study follows the PRISMA guidelines [8]. The screening of articles was conducted through an independent review of titles and abstracts by two authors, followed by the verification of study eligibility, with disagreements resolved via consensus. Subsequently, full texts of the selected articles were thoroughly examined to ascertain study type, imaging details, and outcomes, focusing on early graft occlusion and its factors. For the quality assessment of the included studies, the ROBINS-I tool was utilized to evaluate the risk of bias in non-randomized studies, assessing factors such as confounding, selection bias, and deviations from intended interventions, etc. [9]. No search software was used. The detailed search strategy is reported in the Supplementary Materials.

### 2.1. Inclusion and Exclusion Criteria

**Figure 1:**
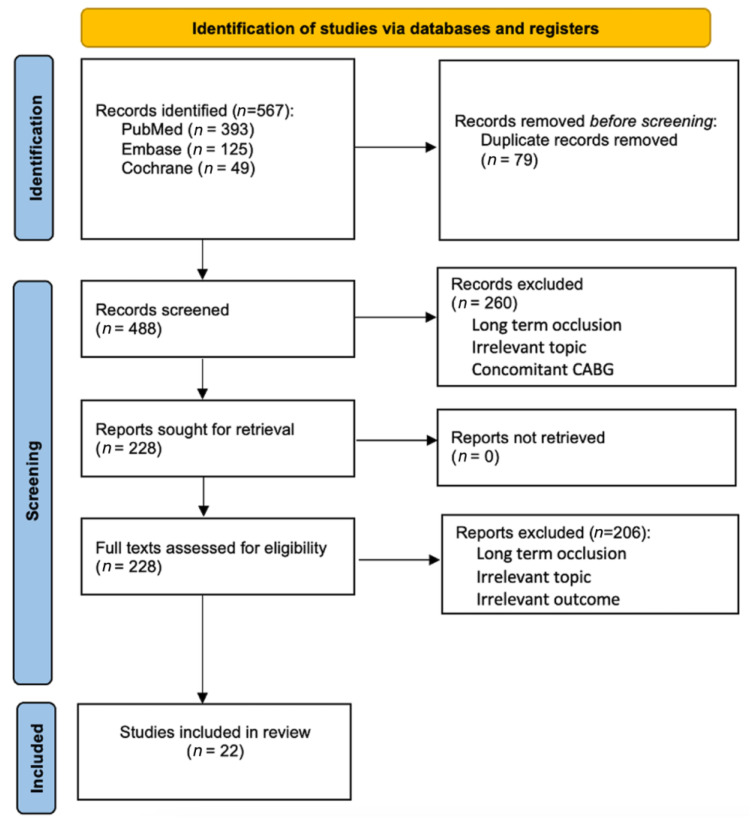
Flow diagram of study selection.

Table 1 outlines the key PECO (Population, Exposure, Comparison, Outcome) elements that guided this systematic review and meta-analysis. Eligible studies were those that concentrated on in-hospital early graft occlusion occurring in the early postoperative period and published since 2004. These studies needed to report on adult patients aged 18 years and older undergoing isolated CABG. We included randomized trials, cohort studies, and observational studies to encompass a broad range of evidence. Both prospective and retrospective studies were considered, particularly those comparing different groups, such as patent versus occluded grafts. We have included both emergency and elective cases, as well as CABG procedures performed on-pump and off-pump, to ensure a comprehensive analysis of early graft occlusion across varying surgical contexts. Additionally, studies were required to employ clear diagnostic criteria for the graft occlusion. Only articles written in English and available in full text were considered. Lastly, a minimum study population of 50 participants was necessary to ensure that the findings were based on a sufficiently large sample to draw meaningful conclusions. Excluded from our review were studies that focused on graft occlusion following non-isolated CABG and studies published before 2004. We also set aside editorials, reviews, case reports, animal studies, and conference abstracts to concentrate solely on original research offering empirical data. Studies that did not present a clear methodology or lacked primary data were deemed unsuitable for our analysis. Excluded were studies that focused on long-term graft occlusions (1–5 years after CABG). Importantly, research centered on early graft occlusions occurring weeks or months post-CABG was also excluded, as our focus was on in-hospital graft failure occurring immediately within days following the procedure. Investigations into genetic factors influencing graft occlusion were excluded, as were studies lacking objective diagnostic criteria (see Figure 1).

### 2.2. Statistical Analysis

Initially, we assessed the quality and consistency of the data extracted from the selected studies. The following data were extracted from the included studies: study characteristics, patients’ characteristics, imaging findings, procedural characteristics, incidence of in-hospital graft occlusion, factors associated with the graft occlusion, and the key findings. We extracted the reported incidence of in-hospital graft occlusion, their 95% CI, and the calculated individual weight of each study. To derive a pooled estimate of the occlusion incidence, we conducted the meta-analysis, including all studies grouped by the type of graft imaging, and visualized the results as a forest plot. A pooled estimate of an event rate is a weighted average of event rates reported in multiple studies, derived using meta-analytic methods with the aim to provide a more realistic and robust incidence rate than an individual study alone. The meta-analysis was executed employing a random-effects inverse-variance model, with the DerSimonian–Laird estimator for between-study variance (τ2) [10]. This model choice accounted for potential variability among study results that may arise from different study designs, populations, and methodologies.

We conducted a quantitative synthesis of data to pinpoint potential risk factors for early graft occlusion post-CABG. A forest plot was created to display the pooled odds ratios and their 95% confidence intervals for various risk factors. The significance of these pooled effect sizes was evaluated using *Z*-tests, considering *p*-values below 0.05 as significant. The heterogeneity between the studies was assessed using a chi square test and expressed as I² statistic. The statistical analysis was performed by a biostatistician (B.G.) using Stata (StataCorp. 2023. Stata Statistical Software: Release 16. College Station, TX, USA: StataCorp LLC.).

## 3. Results

### 3.1. Study Selection

The literature search yielded 567 studies potentially relevant for the inclusion. After this, a total of 79 duplicates were removed. Through the screening of titles and abstracts, we excluded 260 studies, which led us to assess 228 full-text articles for eligibility. Ultimately, 22 studies with 35,798 patients met the inclusion criteria (Figure 1). Most research, including antithrombotic trials, on early graft occlusion after CABG focused on the period from 30 days to 12 months postoperatively and, therefore, were not included in the present systematic review [11,12]. However, our study specifically focused on the detection of early in-hospital graft occlusion immediately following CABG, enabling the prompt implementation of necessary interventions right after surgery.

Table 2 provides an overview of the 22 studies included in this systematic review, highlighting key information such as the type of CABG performed, imaging modalities used, timing of postoperative imaging, and reported occlusion incidence. The studies encompassed in the systematic review spanned a period from 2004 to 2024. The research incorporated a broad range of sample sizes, with the smallest study including 87 participants and the largest encompassing 7461 individuals. Nine studies included a sample size exceeding five hundred patients, while thirteen studies involved fewer than five hundred patients. Among the studies evaluated, fifteen utilized a retrospective design and seven adopted a prospective design. The mean follow-up duration for these studies was 6 ± 5 days. The participants in these studies had a median age of 65 ± 4.8 years, and females constituted 23.4% of the study populations. Geographically, the studies were distributed across various regions, with eight originating from Asia, ten from Europe, and four from North America (Table 2).

A detailed overview of the included studies is presented in the Supplementary Materials. The risk of bias for the included studies was assessed using the ROBINS-I tool, which evaluates seven key domains. Most studies demonstrated a moderate risk of bias, primarily due to potential confounding factors that were not fully adjusted. Despite the use of standardized protocols and objective measurement methods such as coronary angiography or computed tomography angiography in most studies, the incomplete adjustment for clinical and surgical confounders were common concerns. However, the risk of bias due to missing data and deviations from intended interventions was generally low across the studies, as most adhered to their predefined protocols and had minimal loss to follow up. Out of all the assessed studies, only the study by Takahashi et al. explicitly mentioned using blinding in the outcome assessment, while the rest either did not employ blinding or did not explicitly mention it.

### 3.2. Incidence of In-Hospital Graft Occlusion

**Figure 2:**
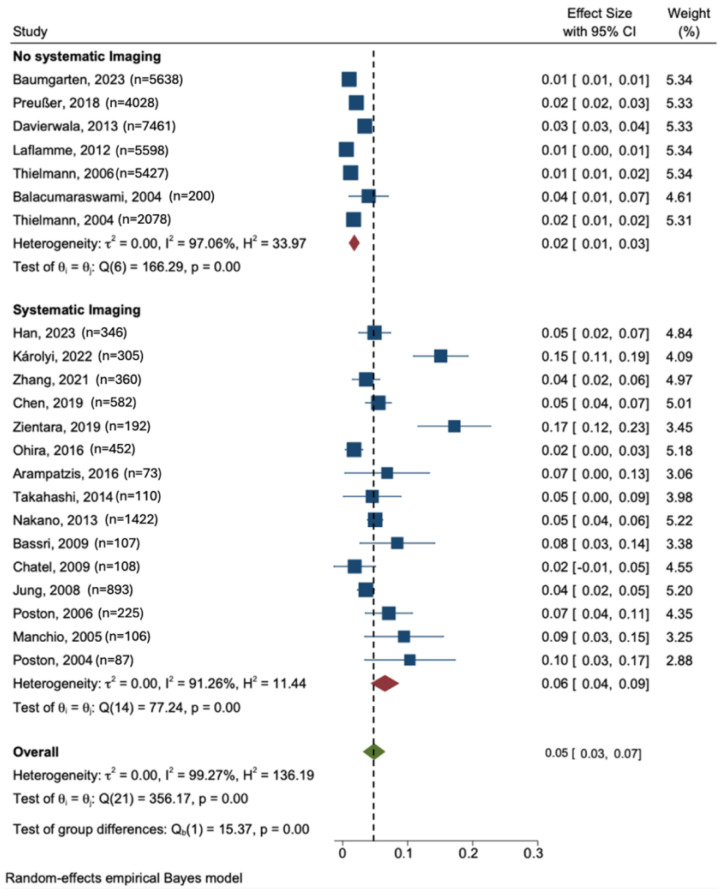
Forest plot comparing the incidence of early graft occlusion in studies utilizing routine vs. non- routine imaging post-CABG. τ^2^ between-study variance in outcome units was <0.005%, estimated using empirical Bayes. I^2^ (%) percentage of total variation that is attributed to heterogeneity across studies rather than chance, range of0–100. Low: 25%, moderate: 50%, and high: 75%. We found 99. H^2^ describes the ratio of the observed variation, measured by Cochran’s Q test, and the expected variance due to sampling error. H^2^ ≤ 1 means no between-study heterogeneity, H^2^ > 1 means there is between-study heterogeneity. p_Q *p* of Cochran’s Q test of residual homogeneity testing the H0 that all effect sizes of the studies are equal. We found *p* < 0.001, indicating unequal effects among studies [1,2,5,6,7,13,14,15,16,17,18,19,20,21,22,23,24,25,26,27,28,29].

In the meta-analysis, the pooled incidence of early graft occlusion was determined to be 5% (95% CI: 3% to 7%) (Figure 2). However, stratification via the use of an imaging approach revealed that studies that used symptom-driven non-routine imaging reported a pooled incidence of early graft occlusion of 2% (95% CI: 1% to 3%). In contrast, studies that implemented routine imaging for all patients documented a pooled incidence at 6% (95% CI: 4% to 9%). Incorporating routine imaging as a covariate in a meta-regression produced an effect size of 0.04 (95% CI: 0.01 to 0.07). Conversely, including a large sample size (N > 500) as a covariate revealed a contrasting effect size of −0.04 (95% CI: −0.07 to −0.01) (Supplementary Materials).

### 3.3. Factors Associated with In-Hospital Graft Occlusion

**Figure 3:**
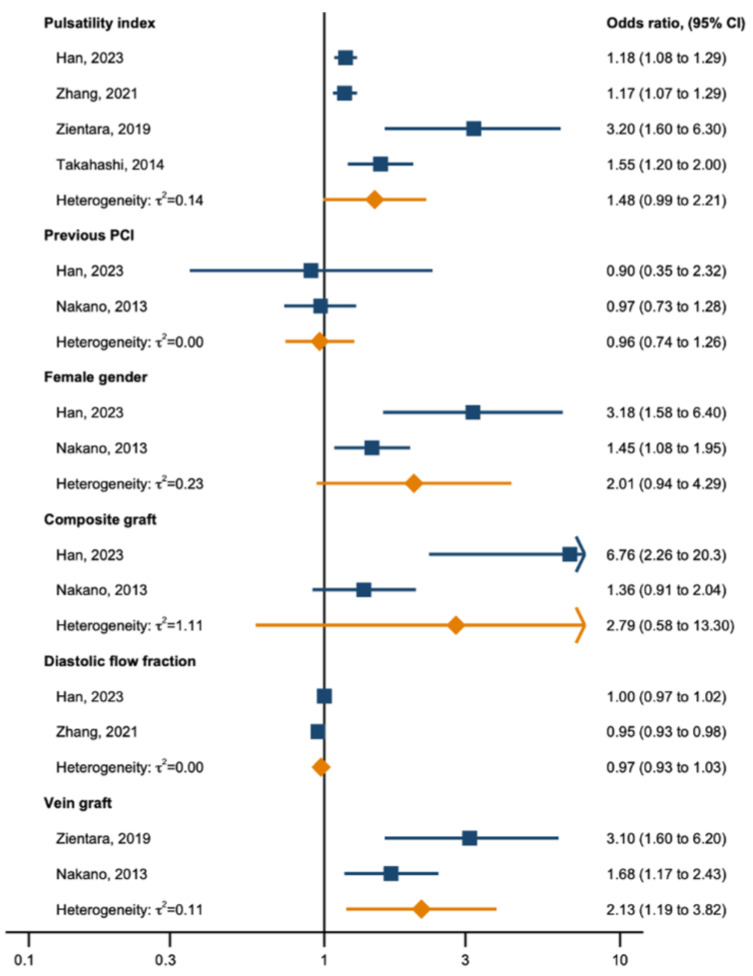
Forest plot of risk factors for early graft occlusion post-CABG. The square markers represent the OR for individual studies, with the size reflecting the study weight in the meta-analysis. The diamond markers represent the pooled ORs for each risk factor, with the width corresponding to the 95% CI. The vertical line denotes an OR of 1, indicating no effect. Values to the right of the line suggest an increased risk of early graft occlusion, while values to the left suggest a decreased risk. Heterogeneity is quantified with I^2^ statistics and *p*-values, assessing the Cochranes heterogeneity test [1,2,6,7,21].

For this meta-analysis, the factors of early graft occlusion were chosen based on their availability across multiple studies to ensure consistent predictors for a meaningful meta-analysis of odds ratios. The pooled risk factors for in-hospital occlusion are presented in Figure 3. The presence of a vein graft was significantly associated with an increased risk of early graft occlusion—OR 2.13, 95% CI: 1.19 to 3.82. Contrastingly, previous PCIs did not show a significant association with early graft occlusion—OR 0.96, 95% CI: 0.74 to 1.26. Female sex—OR 2.01, 95% CI: 0.94 to 4.29—was not a statistically significant factor of early graft occlusion. Composite grafts were not associated with early graft occlusion. The diastolic flow fraction did not present a significant effect—OR 0.97, 95% CI: 0.93 to 1.03. Finally, the results demonstrate a nearly statistically significant association between a higher pulsatility index and early graft occlusion (OR 1.48, 95% CI 0.99 to 2.21). The confidence interval narrowly misses the threshold for statistical significance, highlighting a noteworthy trend that suggests the importance of the pulsatility index in predicting early graft failure.

A funnel plot was used to assess potential publication bias in the meta-analysis. The visual inspection of the funnel plot did not reveal any significant asymmetry, suggesting that the results of this systematic review and meta-analysis are unlikely to be influenced by severe publication bias.

## 4. Discussion

This is the first systematic review and meta-analysis to date on in-hospital early coronary bypass graft occlusion occurring during hospital stay. The meta-analysis revealed a pooled incidence of early graft occlusion of 5%. However, studies that utilized imaging exclusively for patients with symptoms of myocardial ischemia reported an incidence of early graft occlusions of just 2%. In contrast, studies that conducted routine CT imaging for all patients identified a 6% incidence of graft occlusions. This suggests that routine postoperative surveillance could lead to higher reported rates of occlusion, highlighting its benefits in early detection and potentially improving patient outcomes. Our systematic review focuses on graft occlusion occurring during the hospital stay before discharge, in contrast to most research, including antithrombotic trials, which examine occlusion from 30 days to 12 months post-CABG; thus, these studies were excluded [11,12,30].

Previous studies reported female sex and an age above 75 as significant risk factors for early graft occlusion [1,2,7]. In our systematic review, data pertaining to certain risk factors, such as troponin TI and CK-MB levels, were not pooled due to the scant number of studies reporting on these variables. Transitioning to surgery-related factors, the choice of graft material emerges as a critical consideration. The comparison between vein grafts and internal mammary arteries (IMAs), alongside the decision for total arterial revascularization, is paramount [26,31]. IMAs exhibit the lowest occlusion rates, ranging from 1% to 2.5% [26,31]. Conversely, radial arteries also demonstrate enhanced patency compared to saphenous veins, with occlusion rates hovering between 3% and 4% [26,31]. Saphenous veins, however, present the highest occlusion rates, spanning from 3% to 12% [26,31].

Research indicates that surgical factors also play a crucial role in determining graft patency, emphasizing the significance of the graft’s integrity and size and the duration of cardiopulmonary bypass [5,13,19,22,32]. Studies distinctly highlight the nuanced influence of graft material and technique on early graft occlusion, pointing out the protective benefits of sequential grafting and underscoring the critical role of graft size in achieving successful outcomes [2,13,14]. In our analysis, neither previous PCI nor the use of composite grafts were significantly associated with early graft occlusion post-CABG. However, previous research indicates that the review found that the mean flow and pulsatility index were also critical markers of early graft occlusion [2,6,21,33]. Among all the included papers that assessed early graft occlusion days after CABG, only the study by Han evaluated the odds ratio (OR) of antiplatelet therapy [1].

In this meta-analysis, the pooled incidence of early graft occlusion was determined to be 5% (Figure 2). A pooled estimate combines results from multiple studies to provide an overall incidence rate, offering a more reliable estimate than individual studies alone. Stratification by imaging approach revealed that studies using symptom-driven non-routine imaging reported a pooled incidence of early graft occlusion of 2%. In contrast, studies implementing routine imaging for all patients documented a pooled incidence of 6%. Incorporating routine imaging as a covariate in a meta-regression produced an effect size of 0.04.

Among the included studies, those that employed routine imaging reported a higher incidence of occlusion, as they detected more cases, including asymptomatic ones. In contrast, studies that used only symptom-driven imaging diagnosed fewer symptomatic cases. The detected 4% difference in early graft occlusion between studies that report routine and selective graft imaging may be attributed to silent, asymptomatic occlusions [1,2]. Asymptomatic graft occlusions are gaining more recognition in the recent literature, thereby adding a layer of complexity to the interpretation of the early graft occlusion incidence [1,2]. While only a handful of studies have shed light on early asymptomatic graft failures, the implications of such occurrences on patient outcomes cannot be underestimated [1,2]. Early asymptomatic graft failures, though initially undetected, may exert detrimental effects on both short- and long-term patient outcomes [19,34]. These latent complications often manifest when the patient’s physical activity intensifies, leading to symptoms as the myocardial areas, still deprived of adequate blood supply, are subjected to an increased demand [1,2]. Silent early graft occlusion is a stealthy adversary with dire consequences, including myocardial ischemia, which can escalate to urgent re-interventions and compromise long-term survival [1,2]. Hence, the significance of routine identification and addressing asymptomatic early graft occlusion is paramount, not only for the accurate representation in clinical research, but also for the comprehensive management and improved prognosis of patients undergoing CABG.

The heterogeneity in our analysis arises from variations in sample size, study design, diagnostic methods, and imaging timing across the included studies. The majority of data derives from five German studies that used selective CT imaging, which may introduce bias. Differences in imaging techniques and the mix of prospective and retrospective studies further contribute to variability. We chose to accept this heterogeneity to maintain a comprehensive analysis and addressed it through meta-regression rather than excluding studies to create artificial homogeneity.

In our systematic review, quality assessment using the ROBINS-I tool indicated that most studies exhibited a moderate risk of bias, primarily due to confounding factors and a lack of blinding, with some studies showing low risk in areas such as the classification of interventions and deviations from intended interventions. The key bias identified was related to the detection of early graft occlusion, influenced by the diagnostic strategy. Studies using routine CT scans reported higher occlusion rates (around 6%) compared to those scanning only symptomatic patients (about 2%), indicating ascertainment bias. This suggests that universal CT scanning may inflate occlusion rates by detecting both symptomatic and asymptomatic cases, which must be considered when interpreting results.

CTA emerges as a compelling, low-invasive alternative for early graft evaluation post-CABG, offering over 96% sensitivity and specificity for detecting graft failures [1,2] Its adoption in some European hospitals for routine postoperative assessment on the 7th day has unveiled a 17% occlusion rate, highlighting its pivotal role in timely graft patency evaluation and in enhancing surgical planning and outcomes [2]. Routine CT scanning is more likely to identify both symptomatic and asymptomatic occlusions, hence inflating the perceived incidence. In the included studies that employed routine imaging, the imaging was typically performed on average on the 6th day postoperatively.

Cardiovascular imaging, especially advanced echocardiography, plays a critical role in detecting early graft occlusion [35]. Techniques like myocardial work assessment and global longitudinal strain (GLS) are valuable for identifying subclinical myocardial dysfunction before more invasive methods like CTA are needed [35]. Incorporating these advanced tools into routine postoperative care can enable earlier intervention and potentially improve patient outcomes by catching complications sooner [35].

### 4.1. Early vs. Late Graft Occlusions

Most early graft occlusions, occurring within the initial month post-surgery and affecting up to 10% of patients, are predominantly due to mechanical causes [36]. In contrast, late graft stenosis or occlusion, manifesting over a period of 5 to 10 years, predominantly results from an atherosclerotic process, impacting the majority of grafts [36]. While the bulk of existing research has concentrated on mid- to long-term graft occlusion, our study specifically targets early graft occlusion occurring before hospital discharge.

To elucidate the dynamics of EGO in CABG, it is pivotal to compare it with the long-term graft failure. Recently published meta-analysis of individual pooled data of seven trials revealed a notable 33.7% incidence of graft failure after 1-year post-CABG [4]. Factors like age and female sex were also independently linked to increased graft failure, mirroring the factors highlighted in our study [4]. Clinically, graft failure significantly correlated with adverse outcomes, including myocardial infarction, repeat revascularization, and mortality [4]. These findings underscore the grave implications of graft patency for patient outcomes. Therefore, early risk-based diagnosis and tailored interventions are imperative to mitigate the grave consequences and complications associated with graft failure, ultimately enhancing patient outcomes and longevity post-CABG.

### 4.2. Limitations

Our study has several limitations. Firstly, we did not report which symptoms were used to justify symptom-driven imaging, whether it was only chest pain or if there was objective evidence of myocardial ischemia, such as ECG changes or troponin rise. Secondly, the health consequences and management of early graft occlusions were beyond the scope of our study. The included studies did not provide information on the varying severity of consequences for patients with early occlusions. Third, an important limitation is that factors like surgeon experience, graft harvesting technique, the severity of native coronary disease, and the degree of flow limitation, and target vessel quality were not assessed and thus could not be considered as factors associated with early graft occlusion. Another limitation of this study is the lack of data on antiplatelet and anticoagulant intake and its effects on early graft occlusion, as well as the absence of clear reporting on the criteria used to select the type of imaging (CTA or CAG) for assessing graft patency. At the same time, to our knowledge, this is the first systematic review and meta-analysis focusing specifically on in-hospital graft occlusion after CABG. The key strength of this review is its focus on the in-hospital period, providing a comprehensive analysis of early graft occlusion before patient discharge. This immediate postoperative window is critical for identifying and managing complications, and our findings underscore the potential for routine imaging to improve early detection and intervention, thereby potentially enhancing patient outcomes.

In summary, several gaps in the current evidence remain. Key risk factors such as diabetes mellitus, myocardial contractility, and antiplatelet/anticoagulant therapy are often underreported, limiting our understanding of the multifactorial causes of early graft occlusion. Additionally, there is a lack of detailed information on postoperative management strategies and their impact on outcomes, as well as inconsistent reporting of long-term clinical consequences. Future research should focus on comprehensive risk factor analysis, standardized postoperative care protocols, and diverse patient populations to better understand and mitigate the risks associated with early graft occlusion following CABG.

## 5. Conclusions

Silent early graft occlusion can lead to significant complications, including myocardial ischemia and the need for urgent re-interventions, underscoring the importance of early detection and proactive management to safeguard long-term patient outcomes. The findings of this systematic review and meta-analysis underscore the critical importance of routine postoperative imaging to accurately assess early graft occlusion in patients undergoing CABG. The incidence of early graft occlusion appears substantially underestimated when imaging is restricted to symptomatic patients alone. Routine imaging, which detects both symptomatic and asymptomatic occlusions, reveals a higher incidence, emphasizing its role in improving patient outcomes through early intervention. Autoarterial revascularization should be prioritized over autovenous bypass surgery due to its superior outcomes in early graft patency.

## Figures and Tables

**Table 1:** PECO summary.

PECO Element	Description
Population	Patients undergoing CABG.
Exposure	Early postoperative graft imaging after CABG.
Comparison	Patients with occluded grafts vs. patients with patent grafts.
Outcome	Primary: Incidence of early graft occlusion. Secondary: Risk factors for early graft occlusion.

**Table 2:** Overview of 22 studies included into the systematic review and meta-analysis.

Author(s)	Year	Country	Study Design	Sample Size	Age M ± SD/(IQR)	Sex (Female)	Modality of Diagnosis	PostOP Imaging (days)	Occlusion	Overall Risk of Bias
Poston, et al. [13]	2006	USA	Prospective	225	69 ± 12	34%	CTA	5	7%	Moderate
Han, et al. [1]	2023	China	Retrospective	346	64 (57–69)	28%	CTA	4–30 (prior to discharge)	5%	Moderate
Zientara, et al. [2]	2019	Switzerland	Retrospective	192	66 (59–73)	19%	CTA	7	17.2%	Moderate
Nakano, et al. [7]	2013	Japan	Retrospective	1422	68 ± 9	25%	CAG+MDCT	14	5.0%	Moderate
Manchio, et al. [14]	2005	USA	Prospective	106	68 ± 10	37%	CTA	5	9%	Moderate
Jung, et al. [15]	2009	Republic of Korea	Retrospective	893	62 ± 8	25%	CTA	7	3.6%	Moderate
Balacumaraswami, et al. [16]	2004	UK	Prospective	200	63 ± 10	N/A	Fluorescence Imaging	Intraoperative	4.0%	Moderate
Arampatzis, et al. [17]	2016	Greece	Prospective	73	65 ± 9	22%	MSCT	5	7%	Moderate
Zhang, et al. [6]	2021	China	Retrospective	360	64 (57–69)	27%	CTA	N/A (prior to discharge)	3.50%	Moderate
Baumgarten, et al. [5]	2023	Germany	Retrospective	5638	68 ± 9	21%	CAG	N/A (prior to discharge)	1%	Moderate
Poston, et al. [18]	2004	USA	Prospective Randomized trial	87	N/A	N/A	CTA	5	10.30%	Moderate
Bassri, et al. [19]	2009	Iran	Prospective	107	60 ± 9	24%	MSCT	7	8.70%	Moderate
Thielmann et al. [20]	2004	Germany	Prospective	2078	65 ± 9	27%	CAG	1	1.70%	Moderate
Takahashi et al. [21]	2014	Japan	Retrospective	110	68 (34–89)	17%	CAG	Intraoperative	4.70%	Low
Preußer, et al. [22]	2018	Germany	Retrospective	4028	67 ± 10	25%	CAG	1	2%	Moderate
Chatel, et al. [23]	2009	France	Retrospective	108	64.9	9.3%	CAG	8 days	2%	Moderate
Károlyi, et al. [24]	2022	Switzerland	Retrospective	305	68	12%	CTA	6 days	15%	Moderate
Ohira, et al. [25]	2016	Japan	Retrospective	452	65.7	16.6%	CAG and CTA	N/A (prior to discharge)	1.7%	Moderate
Laflamme, et al. [26]	2012	Canada	Retrospective	5598	65.8	31%	CAG	3 days	1%	Moderate
Chen, et al. [27]	2019	China	Retrospective	582	61.5	21.5%	MSCTA	5 days	5%	Moderate
Davierwala, et al. [28]	2013	Germany	Retrospective	7461	67.8	22.6%	CAG	3 days	3%	Moderate
Thielmann, et al. [29]	2006	Germany	Retrospective	5427	66 ± 7	N/A	CAG	1 day	1%	5

CAG—coronary angiography; CTA—computed tomography angiography; MSCT—Multi-Slice Computed Tomography; MDCT—Multi-Detector Computed Tomography.

## Data Availability

The data underlying this article are available in the article and in its online Supplementary Materials.

## Supplementary Materials

The following supporting information can be downloaded at https://www.mdpi.com/article/10.3390/jcm13185514/s1, Table S1: Overview of study characteristics; Table S2: Meta-Regression.
