# Supplementary material for: In-Hospital Graft Occlusion in Post-Coronary Artery Bypass Grafting Patients in the Early Postoperative Period: A Systematic Review and Meta-Analysis
Source: J Clin Med. 2024 Sep 18;13(18):5514. doi: 10.3390/jcm13185514 (PMC11432121; doi:10.3390/jcm13185514)
Supplement: Supplementary file 1 [file jcm-13-05514-s001.zip › Supp material 1.pdf]

SEARCH STRATEGY

Pubmed

("Coronary Artery Bypass"[Mesh] OR CABG OR "coronary artery bypass graft" OR "coronary artery bypass grafting" OR "coronary bypass" OR "heart bypass surgery") AND (("early" AND ("graft failure" OR "graft occlusion" OR "graft closure" OR "vascular occlusion" OR "postoperative occlusion" OR “graft thrombosis” OR “coronary occlusion”)) OR ("early graft failure" OR "early graft occlusion" OR "early graft closure" OR "early vascular occlusion" OR "immediate graft failure" OR "early postoperative occlusion" OR “early graft thrombosis”))

Embase

('coronary artery bypass'/exp OR CABG OR 'coronary artery bypass graft' OR 'coronary artery bypass grafting' OR 'coronary bypass' OR 'heart bypass surgery') AND (('early' NEAR/3 ('graft failure' OR 'graft occlusion' OR 'graft closure' OR 'vascular occlusion' OR 'postoperative occlusion' OR 'graft thrombosis' OR 'coronary occlusion')) OR ('early graft failure' OR 'early graft occlusion' OR 'early graft closure' OR 'early vascular occlusion' OR 'immediate graft failure' OR 'early postoperative occlusion' OR 'early graft thrombosis'))

Cochrane

("Coronary Artery Bypass"[MeSH] OR CABG OR "coronary artery bypass graft" OR "coronary artery bypass grafting" OR "coronary bypass" OR "heart bypass surgery") AND (("early" NEAR/3 ("graft failure" OR "graft occlusion" OR "graft closure" OR "vascular occlusion" OR "postoperative occlusion" OR "graft thrombosis" OR "coronary occlusion")) OR ("early graft failure" OR "early graft occlusion" OR "early graft closure" OR "early vascular occlusion" OR "immediate graft failure" OR "early postoperative occlusion" OR "early graft thrombosis"))

INCLUSION AND EXCLUSION CRITERIA

INCLUSION

1. **Study Population:**

   Adult patients aged 18 years and older.

   Patients who have undergone isolated CABG surgery

2. **Outcome of Interest:**

Studies must report on early graft occlusion, as a primary or secondary endpoint
Early graft occlusion should be defined as occlusion occurring before hospital discharge or within a specified early postoperative period (e.g., within 30 days of surgery).

3. Study Design:
Randomized controlled trials, cohort studies, casecontrol studies, and observational studies.
Both prospective and retrospective studies.
Studies performing comparison between groups, e.g. patent vs occluded

4. Predictors Investigated:
Studies focusing on identifying factors associated with early graft occlusion (e.g., comorbidities, age, sex), surgical techniques, intraoperative factors, and postoperative care.

5. Diagnostic Criteria:
Studies must use clear and recognized diagnostic criteria for graft occlusion (e.g., CT angiography, Doppler ultrasound findings).

6. Language and Availability:
Studies published in English.
Fulltext articles available.

7. Sample size
Minimum of 50 patients

EXCLUSION

1. Study Population:
Studies involving pediatric patients (younger than 18 years).
Patients undergoing CABG as part of a combined surgical procedure.

2. Outcome of Interest:

Studies not specifically reporting on early graft occlusion as defined by the above criteria.
Studies focusing on longterm graft patency beyond the early postoperative period.

3. Study Design:

Editorials, reviews, case reports, animal studies, and conference abstracts.
Studies without a clear methodology or lacking primary data.
Investigation of long-term graft occlusion

4. Predictors Investigated:

Studies that investigate genetic factors (eg.gene expression)

5. Diagnostic Criteria:

Studies using nonstandard or vague definitions of graft occlusion.
Studies lacking objective diagnostic criteria.

6. Language and Availability:

NonEnglish studies, unless translation is feasible and reliable.
Inaccessible studies (without available full text).
Studies published before 2004
