# Supplementary material for: In-Hospital Graft Occlusion in Post-Coronary Artery Bypass Grafting Patients in the Early Postoperative Period: A Systematic Review and Meta-Analysis
Source: J Clin Med. 2024 Sep 18;13(18):5514. doi: 10.3390/jcm13185514 (PMC11432121; doi:10.3390/jcm13185514)
Supplement: Supplementary file 1 [file jcm-13-05514-s001.zip › Supp material 2.pdf]

TABLE S1. Overview of study characteristics

																	Newcastle-Ottawa Scale							
																	Selection				Comparability	Outcome			Total
Title	doi link	Author(s)	Year of Publication	Country	Study Design	Sample Size	Age	Gender (Female)	On/Off-pump CABG	Systematic Imaging	PostOP Imaging (mean)	% Occlusion	Predictors of Early Occlusion: OR (p<0,05)	Non-significant predictors of occlusion	Imaging Method	Key Finding	Representativeness of the exposed cohort	Selection of the non-exposed cohort	Ascertainment of exposure	Demonstration that outcome of interest was not present at start of study	Comparability of cohorts based on the design or analysis controlled for confounders	Assessment of outcome	Was follow-up long enough for outcomes to occur	Adequacy of follow-up of cohorts	Out of 9
**Endothelial injury and acquired aspirin resistance as promoters of regional thrombin formation and early vein graft failure after coronary artery bypass grafting**	10.1016/j.jtcvs.2005.08.058	Poston, et al	2006	USA	Prospective Cohort	225	69±12	34%	off	1	5 days	7%	Endothelium cell disruption in the SV: 5.98 (95% CI 2.15-10.46) Aspirin resistance on day 1: 2.59 (95% CI 1.13-5.95) Target diameter: 0.035 (95% CI 0.002-0.734)	N/A	CTA	Insufficient suppression of coagulation from aspirin (aspirin resistance) developing early after OPCAB and EC disruption in the SVG synergistically increased the risk of early graft thrombosis.	*		*	*	*	*	*	*	7
**Early asymptomatic graft failure in coronary artery bypass grafting:**	10.1186/s13019-023-02199-0	Han, et al	2023	China	Retrospective Cohort	346	64 (57–69)	28%	Mixed	1	4-34 days	5%	Female gender (OR 3.181, CI 1.58–6.40) Composite grafting (OR 6.762, CI 2.26–20.28)	Previous PCI (OR 0.9, CI 0.35-2.32) Diastolic flow fraction (OR 1.0,	CTA	Early asymptomatic graft failure is associated with both patient and surgical factors including	*		*	*	*	*	*	*	7

**a study based on computed tomography angiography analysis**													PI (OR 1.180, CI 1.08–1.29) PostOP atrial fibrillation (OR 2.348, CI 1.15–4.78)	CI 0.97-1.02) In situ IMAs (OR 0.47, CI 0.2-1.21)		female gender, high PI value, composite graft strategy and the new POAF. However, the early postoperative dual-antiplatelet ther- apy with aspirin and clopidogrel may be useful for pre- venting graft failure.								
**Early silent graft failure in off-pump coronary artery bypass grafting: a computed tomography analysis**	10.1093/ejcts/ezz112	Zientara, et al	2019	Switzerland	Retrospective Cohort	192	66 (59-73)	19%	off	1	7 days	17,2%	Decreased Intraoperative flow (OR 2.3, CI 1.1–4.3) PI (OR 3.2, CI 1.6–6.3) Use of venous grafts (OR 3.1, CI 1.6–6.2)	N/A	CTA	Early silent graft failure occurred predominantly in venous grafts with a tendency to female gender. Lower flow rate and higher pulsatility index were significantly associated with graft occlusion.	*	*	*	*	*	*	*	7
**Early angiographic evaluation after off-pump coronary artery**	10.1016/j.jtcvs.2012.08.057	Nakano, et al	2013	Japan	Retrospective Cohort	1422	68 ± 9	25%	off	1	14 days	5,0%	Operation time (min) (OR 1.13, CI 1.01-1.27) SVG (OR 1.68, OR 1.17-2.43) Recipient coronary	" Age >75 y (OR 1.39, CI 1.02-1.90) Female sex (OR 1.45, CI 1.08-	Catheter-based angiography +MDCT	Severe percent stenosis diameter and small recipient cor- onary diameter were	*	*	*	*	*	*	*	7

**bypass grafting**		stenosis >75% (OR 0.71, CI 0.53-0.93)<br>Recipient coronary diameter < 1.5 mm (OR 1.61, CI 1.25-2.09)<br>Max CK-MB >50 IU/L (OR 1.66, CI 1.12-2.47)<br>Sequential graft (OR 0.69, CI 0.51-0.94)	1.95)<br>NYHA class > 3 (OR 0.88, CI 0.62-1.25)<br>Unstable angina (OR 0.81, CI 0.57-1.16)<br>Emergency (OR 0.86, CI 0.50-1.48)<br>Previous MI (OR 0.99, CI 0.75-1.30)<br>Recent MI (<90 d) (OR 1.21, CI 0.76-1.82)<br>Fair (LVEF > 50%) (OR Reference)<br>Moderate (LVEF 30%-50%) (OR 0.75, CI 0.52-1.07)<br>Poor (LVEF < 30%)	associated with graft patency in OPCAB surgery. A better patency rate would be expected when anastomosing the LITA to a coronary artery with se- vere stenosis.	

(OR 0.93, CI 0.38-2.25)<br>1- or 2-vessel disease (OR Reference)<br>3-vessel disease (OR 1.20, CI 0.58-2.48)<br>Left main disease (OR 0.69, CI 0.51-0.91)<br><br>Previous PCI (OR 0.97, CI 0.73-1.28)<br><br>Previous cardiac surgery (OR 1.64, CI 0.81-3.35)<br>CRF (creatinine > 200 umol/L) (OR 0.61, CI 0.31-1.19)<br>ESRF on dialysis (OR not provided

) Diabetes mellitus (OR 1.04, CI 0.79-1.38) Taking insulin (OR not provided) Hypertension (OR 0.93, CI 0.70-1.23) Hypercholesterolemia (OR 1.07, CI 0.81-1.42) COPD (OR 1.16, CI 0.60-2.26) Extracardiac arteriopathy (OR 0.85, CI 0.61-1.19) CVA (OR 1.04, CI 0.71-1.51) No. of anastomoses (OR 1.01, CI 0.88-1.16) Incomplete

revascularization (OR 0.89, CI 0.57-1.39)<br>LITA (OR Reference)<br>RITA (OR 1.04, CI 0.73-1.48)<br>GEA (OR 1.27, CI 0.82-1.95)<br>Radial artery (OR 0.95, CI 0.29-3.11)<br>LAD (OR Reference)<br>LCx (OR 1.20, CI 0.87-1.65)<br>RCA (OR 1.39, CI 0.99-1.94)<br>Sequential graft (OR 0.80, CI 0.61-1.04)<br>Composite graft (OR 1.36, CI 0.91-2.04)<br>Endarter

														ectomy (OR 2.23, CI 0.65-7.68) Proximal anastomotic devices (OR 0.46, CI 0.20-1.06) Max CK-MB IU/L (OR 1.02, CI 1.00-1.03)"									
**Disruption of Graft Endothelium Correlates With Early Failure After Off-Pump Coronary Artery Bypass Surgery**	10.1016/j.athoracsur.2004.12.054	Manchio, et al	2005	USA	Prospective Cohort	106	68 ± 10	37%	off	1	5 days	9%	N/A		CTA	Trauma to the saphenous vein before grafting, evidenced by endothelial disruption, was strongly associated with early graft thrombosis.	*	*	*	*	*	*	*	7
**Comparison of radial artery patency according to proximal anastomosis site: Direct aorta to radial**	doi:10.1016/j.jtcvs.2008.12.004	Jung, et al	2008	Republic of Korea	Retrospective Cohort	893	62±8	25%	Mixed	1	7 days	3,6%	N/A	N/A	CTA	Direct aorta to RA bypass grafting results in significantly superior early patency rates compared with composite grafting,	*	*	*	*	*	*	*	7

**artery anastomosis is superior to radial artery composite grafting**																							
**Does off-pump total arterial grafting increase the incidence of intraoperative graft failure?"**	10.1016/j.jtcvs.2003.11.072.	Balacumaraswami, et al	2004	UK	Prospective Cohort	200	63±10	N/A	Mixed	0	Intraoperative	4,0%	N/A	N/A	intraoperative imaging system, SPY (Novadaq Technologies Inc), based on the fluorescent properties of indocyanine green dye.	No difference in the incidence of failed grafts when between on-pump and off-pump total arterial grafting.	*	*	*		*	*	5
**Graft failure prior to discharge after coronary artery bypass surgery: a prospective single-centre study using dual 64-slice computed**	10.4244/EIJV12I8A1	Arampatzis, et al.	2016	Greece	Prospective Cohort	73	65±9	22%	Mixed	1	5 days	7%	Odds ratios N/A Arterial disease distal to the anastomosis Radial artery Grafted artery obtuse marginal Previous myocardial infarction Triple-vessel coronary disease	N/A	MSCT	In-hospital acute graft failure rate was 3.4% for grafts and 6.8% for patients, which was modulated by anatomical or procedural characteristics.	*	*	*	*	*	*	6

**tomography**																								
**The predictive value of intraoperative transit-time flow measurement parameters for early graft failure in different target territories**	10.1016/j.jjcc.2020.09.003.	Zhang, et al	2021	China	Retrospective Cohort	360	64 (57-69)	27%	Mixed	1	N/A (prior to discharge)	3,50%	Pulsatility index (OR 1.17, CI 1.07-1.29) Diastolic Flow Fraction (OR 0.95, CI 0.93-0.98)	N/A	CTA	High PI value is a predictive parameter for graft failure not only in overall grafts but also in grafts of different target territories. DF might be more useful for evaluating the quality of grafts in left than in right target territories.	*	*	*	*	*	*		6
**Outcomes After Early Postoperative Myocardial Infarction Due to Graft Failure in Patients Undergoing Coronary Artery Bypass Grafting.**	N/A	Baumgarten, et al	2023	Germany	Retrospective Cohort	5638	68±9	21%	Mixed	0	N/A (prior to discharge)	1%	N/A	N/A	CAG	Clinically apparent EGF is rare, but imaging is mandatory among these patients. EGF is an independent predictor of mortality.	*		*	**	*	*		6
**Virchow Triad but Not Use of an Aortic Connector**	10.1532/HSF98.20041090.	Poston, et al	2004	USA	A combination of Prospective Cohort	87	N/A	N/A	off	1	5 days	10,30%	N/A	N/A	CTA	Hypercoagulability, as determined by postoperative in vitro	*	*	*	**	*	*	*	8

**Device Predicts Early Graft Failure after Off-Pump Coronary Bypass**					Study and Prospective Randomized Trial											platelet hyperreactivity, was significantly associated with early graft thrombosis							
**Evaluation of early coronary graft patency after coronary artery bypass graft surgery using multislice computed tomography angiography.**	10.1186/1471-2261-9-53.	Bassri, et al	2009	Iran	Prospective Cohort	107	60 ± 9	24%	Mixed	1	7 days	8,70%	N/A	N/A	MSCT	Saphenous vein grafts had an overall patency rate of 90%, and arterial grafts were patent in 95% of the cases.	*	*	*	*	*	*	6
**Role of troponin I myoglobin and creatine kinase for the detection of early graft failure following coronary artery**	10.1016/j.ejcts.2004.03.015	Thielmann et al	2004	Germany	Prospective Cohort	2078	65 ± 9	27%	N/A	0	1 day	1,70%	N/A	N/A	Angiography	Cardiac troponin I (cTnI) is indicated as the best non-invasive diagnostic discriminator for early graft failure after CABG, with an optimal cut-off value 24 hours after aortic unclamping.	*		*	*	*	*	5

**bypass grafting.**																								
**Real-time graft flow assessment using epigraftic ultrasonography during coronary artery bypass grafting.**	10.1093/ejcts/ezt627.	Takahashi et al	2014	Japan	Retrospective Cohort	110	68 (34-89)	17%	Mixed	1	Intraoperatively + early postOp	4,70%	Peak diastolic velocity (OR 0.86, CI 0.78–0.95) Mean diastolic velocity (OR 0.76, CI 0.64–0.91) Diastolic velocity time integral (OR 0.49, CI 0.31–0.76) Systolic velocity time integral (OR 0.52, CI 0.30–0.92) Mean velocity (OR 0.86, CI 0.76–0.96) Pulsatility Index (OR 1.55, CI 1.20–2.00) Percentage of diastolic reverse flow (OR 1.27, CI 1.14–1.41) Percentage of systolic reverse flow (OR 1.19, CI 1.09–1.29)	N/A	CAG	Epigraftic ultrasonography is a useful tool for predicting early graft failure during CABG.Systolic and diastolic reverse flow, and PI can predict early graft failure.	*	*	*	*	*	*	*	7

**Survival results of postoperative coronary angiogram for treatment of perioperative myocardial ischaemia following coronary artery bypass grafting: a single-centre experience**	10.1093/icvts/ivx317	Preußer, et al	2018	Germany	Retrospective Cohort	4028	67±10	25%	Mixed	0	1 day	2%	N/A	N/A	Coronary angiogram	Early PostOp coronary angiogram and treatment of causes underlying PMI may significantly attenuate the detrimental effects of myocardial ischaemia and improve the outcome of these patients.	*		*	**	*	*	*	7
**Impact of purely internal thoracic artery T-graft technique on the mode and quality of surgical myocardial revascularization evaluated by early postoperative coronar**	10.1016/j.acvd.2009.06.008	Chatel, et al	2009	France	Retrospective Cohort	108	64.9	9.3%	N/A	1	8 days	2%			CAG	Exclusive use of the internal thoracic artery T-graft technique achieved nearly complete and effective revascularization, with 98% patency rate of the anastomoses and segments.	*	*	*	*	*	*	*	7

**y angiography**																						
**Routine early postoperative computed tomography angiography after coronary artery bypass surgery: clinical value and management implications**	10.1093/ejcts/ezab390	Károlyi, et al	2022	Switzerland	Retrospective cohort study	305	68	12%	Mixed	1	6 days	15%	CTA	The study found a significant difference in the patency rates between arterial and venous grafts.	*	*	*	*	*	*	*	7
**Safety and Efficacy of Sequential Left Internal Thoracic Artery Grafting to Left Circumflex Area**	10.1016/j.athoracsur.2016.02.075	Ohira, et al	2016	Japan	Retrospective cohort study	452	65.7	16.6%	Mixed	1	N/A (prior to discharge)	1.7%	CAG and CTA	In situ sequential LITA grafting provides acceptable early graft patency and freedom from repeat revascularization, resulting in excellent survival.	*	*	*	*	*		*	6
**Management of early postoperative coronary artery bypass graft failure**	10.1093/icvts/ivr127	Laflamme, et al	2012	Canada	Retrospective cohort study	5598	65.8	31%	N/A	0	3 days	1%	CAG	Early reintervention (PCI or redo-CABG) may limit the extent of myocardial cellular damage compared with	*	*		*	*		*	5

															conservative medical strategy in patients with myocardial ischaemia due to early graft failure.							
**Does Early Graft Patency Benefit from Perioperative Statin Therapy? A Propensity Score-Matched Study of Patients Undergoing Off-Pump Coronary Artery Bypass Surgery**	10.1155/2019/1582183	Chen, et al	2019	China	Retrospective cohort study	582	61.5	21.5%	off	1	5 days	5%	Lower risk of graft occlusion among smokers: OR 0.41, 95% CI, 0.20 to 0.86, p=0.026	MSCTA	Perioperative continuation of statin therapy did not significantly improve early graft patency. A lower risk of graft occlusion was observed among smoking patients who continued statin therapy.	*	*	*	*	*	*	*	7
**Impact of Expeditious Management of Perioperative Myocardial Ischemia in Patients Undergoing Isolated Coronar**	10.1161/CIRCULATIONAHA.112.000347	Davierwala, et al	2013	Germany	Retrospective Cohort	7461	67.8	22.6%	Mixed	0	3 days	3%		CAG	Expeditious management of bypass graft failure results in similar midterm survival to nonischemic patients in hospital survivors. PMI is associated with increased in-hospital	*	*		*	*		*	5

**y Artery Bypass Surgery**															mortality in patients undergoing isolated CABG.						
**Emergency re-revascularization with percutaneous coronary intervention, reoperation, or conservative treatment in patients with acute perioperative graft failure following coronary artery bypass surgery**	10.1016/j.ejcts.2006.03.062	Thielmann, et al	2006	Germany	Retrospective Cohort	5427	66±7	N/A	N/A	0	1 day	1%		CAG	Re-revascularization with emergency PCI may limit the extent of myocardial cellular damage compared with the surgical-based treatment strategy in patients with acute perioperative myocardial ischemia due to early graft failure following CABG.	*	*	*	*	*	5

TABLE S2. META REGRESSION

	Coefficient (95% CI)	*p*	R^2	*p_Q_b*	τ^2	I^2	H^2
Systematic Imaging	0.04 (0.01 to 0.07)	0.004	33	<0.001	0	99	94
N>500	-0.04 (-0.07 to -0.01)	0.009	28	<0.001	0	99	101
Retrospective	-0.02 (-0.06 to 0.02)	0.332	0	<0.001	0	99	134

Coefficient (95% CI)' - the effect size with its confidence interval for systematic graft imaging and study size (n>500). 'P' values show the significance of these effects. R^2 measures the variance explained by the model, and I^2 and H^2 quantify heterogeneity across the included studies.
